# Rethinking Therapeutic Strategies for Anorexia Nervosa: Insights From Psychedelic Medicine and Animal Models

**DOI:** 10.3389/fnins.2020.00043

**Published:** 2020-02-04

**Authors:** Claire J. Foldi, Paul Liknaitzky, Martin Williams, Brian J. Oldfield

**Affiliations:** ^1^Department of Physiology, Monash University, Clayton, VIC, Australia; ^2^Monash Biomedicine Discovery Institute, Clayton, VIC, Australia; ^3^Faculty of Health, Deakin University, Burwood, VIC, Australia; ^4^Monash Institute of Pharmaceutical Sciences, Parkville, VIC, Australia; ^5^Psychedelic Research in Science and Medicine Inc., Melbourne, VIC, Australia

**Keywords:** anorexia nervosa, psychedelic medicine, psilocybin, serotonin, 5-HT_2__A_, animal models, activity-based anorexia, cognitive flexibility

## Abstract

Anorexia nervosa (AN) has the highest mortality rate of any psychiatric disease, yet available pharmacological treatments are largely ineffective due, in part, to an inadequate understanding of the neurobiological drivers that underpin the condition. The recent resurgence of research into the clinical applications of psychedelic medicine for a range of mental disorders has highlighted the potential for classical psychedelics, including psilocybin, to alleviate symptoms of AN that relate to serotonergic signaling and cognitive inflexibility. Clinical trials using psychedelics in treatment-resistant depression have shown promising outcomes, although these studies are unable to circumvent some methodological biases. The first clinical trial to use psilocybin in patients with AN commenced in 2019, necessitating a better understanding of the neurobiological mechanisms through which psychedelics act. Animal models are beneficial in this respect, allowing for detailed scrutiny of brain function and behavior and the potential to study pharmacology without the confounds of expectancy and bias that are impossible to control for in patient populations. We argue that studies investigating the neurobiological effects of psychedelics in animal models, including the activity-based anorexia (ABA) rodent model, are particularly important to inform clinical applications, including the subpopulations of patients that may benefit most from psychedelic medicine.

## The Resurgence of Psychedelic Medicine and Therapeutic Potential for AN

Psychedelics were first investigated as therapeutic agents for mental disorders in the 1950s (Neill, 1987) and more than 1000 clinical papers were published on classical psychedelics between 1950 and the mid-1960s (Grinspoon and Bakalar, 1981). However, political concerns over widespread non-clinical use and governmental interventions associated with an emerging counter culture led to regulatory obstacles and an abrupt end to this promising research. Recently, a resurgence of research into the clinical application of psychedelics has emerged (Strassman et al., 1994; Sessa, 2012; Nichols, 2016) and clinical trials have already highlighted psychedelic medicine as a promising alternative to conventional methods in what is being hailed as a “paradigm shift” for the treatment of psychiatric disorders, including depression, post-traumatic stress and substance use disorders (De Gregorio et al., 2018; Schenberg, 2018). In addition, the U.S. Food and Drug Administration (FDA) have twice designated psilocybin for treatment-resistant depression as a “breakthrough therapy,” in 2018 and 2019. Combined with specialized psychotherapy, psilocybin has been shown to decrease symptoms of anxiety and depression that accompany life-threatening cancer diagnoses (Griffiths et al., 2016; Ross et al., 2016), alleviate symptoms in patients with treatment-resistant depression (Carhart-Harris et al., 2016a, 2018a) and improve adherence to abstinence regimes in nicotine-dependent smokers (Johnson et al., 2017). Moreover, patients with obsessive-compulsive disorder (OCD) have shown short-term improvements following psilocybin treatment (Moreno et al., 2006).

Multiple studies investigating the safety and efficacy of psilocybin for the treatment of major depressive disorder have recently commenced across the U.S. and Europe (Clinical Trials: NCT03866174; NCT03775200; NCT03429075; NCT03715127; NCT03181529; NCT03554174; NCT03380442). Importantly, the first Phase 1 study exploring the safety and efficacy of psilocybin in patients with AN was launched in 2019 to examine a range of outcome measures including self-reported anxiety, depression and quality of life as well as changes in body mass index (BMI) and food preference (Clinical Trial: NCT04052568). The findings from this trial will indicate efficacy one way or the other; however, understanding the biological mechanisms that underpin any effects of psilocybin on AN await carefully controlled clinical and animal-based studies. It should be noted that preliminary support for the efficacy of psychedelics on eating disorder symptoms has been shown in qualitative interviews with patients following the ceremonial consumption of ayahuasca, another serotonergic psychedelic (Renelli et al., 2018).

**FIGURE 1:**
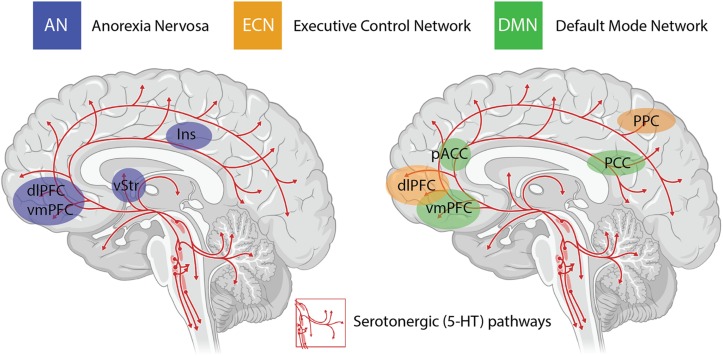
Serotonergic (5-HT) pathways within the brain and the considerable overlap between brain regions implicated in anorexia nervosa (AN), the task-positive executive control network (ECN) and the task-negative default mode network (DMN). These networks are highly interactive and are modulated by activity in the insula (Ins). Changes in functional connectivity within and between these networks are proposed to underlie the impact of psilocybin on cognitive flexibility in AN. dlPFC = dorsolateral prefrontal cortex; vmPFC = ventromedial prefrontal cortex; vStr = ventral striatum; pACC = perigenual anterior cingulate cortex; PCC = posterior cingulate cortex; PPC = posterior parietal cortex.

An important consideration when interpreting the findings from clinical trials using psychedelics is the inability to circumvent certain methodological biases. Even when niacin or very low dose psychedelics are used as active placebos, most participants and therapists are quickly unblinded to the condition, potentially resulting in expectancy biases. The subjective scales used to measure the efficacy of psilocybin in some studies introduces further bias in the assessment of outcome measures (Muttoni et al., 2019). Irrespective of these limitations, the neurobiological “mechanisms” underlying the efficacy of psychedelics for treatment-resistant depression are beginning to be elucidated in very broad terms through administration of psilocybin in combination with brain imaging techniques (Carhart-Harris et al., 2012, 2017). The insights with the most experimental support include alterations in activity and functional connectivity within the default mode network (DMN) (Carhart-Harris et al., 2012, 2013), a group of neural structures that represent resting-state cognition (Raichle et al., 2001; Greicius et al., 2003) (depicted in Figure 1). Functional connectivity between the DMN and other resting state networks is generally shown to increase following administration of psilocybin (Roseman et al., 2014), and unusual levels of co-activation between DMN and task-positive structures has been found under psilocybin (Carhart-Harris et al., 2012, 2013) and ayahuasca (Palhano-Fontes et al., 2015). The mechanisms underlying the action of psilocybin on functional activity in resting state networks remain poorly understood, particularly the role of the serotonergic system, considering that the major midbrain nucleus in which 5-HT cell bodies lie (raphe nucleus; Figure 1) is not included in canonical resting state networks. Regardless, mechanistic models have been developed that assert the action of psychedelics as relaxing high-level prior beliefs and liberating bottom-up information flow (Carhart-Harris and Friston, 2019), and progress is being made to explain the action of psychedelics in the context of complicated neurotransmitter pharmacology, molecular pathways, and plastic changes that contribute to the central actions of these compounds (Ly et al., 2018).

Anorexia nervosa (AN) has the highest mortality rate of any psychiatric disorder (Harris and Barraclough, 1998) and is characterized by a relentless pursuit of weight loss despite severe emaciation. The majority of patients with AN also engage in excessive physical exercise and other compulsive locomotor strategies to avoid or counteract weight gain (Davis, 1997). A number of brain imaging studies in AN point to a combination of decreased neural activity in ventral reward regions (ventral and dorsal striatum) and increased neural activity in prefrontal control regions (orbital and dorsolateral prefrontal cortices) (Kaye et al., 2013). This imbalance may underlie the rigid adherence to punishing diet and exercise regimes by patients with AN that is characterized by both excessive behavioral control and diminished cognitive flexibility (Tchanturia et al., 2004, 2011, 2012). Despite the high mortality and the array of pharmacological and psychotherapeutic strategies that have been employed to treat AN (Davis and Attia, 2017), up to 50% of AN patients suffer with chronic, often life-long illness (Pike, 1998), indicating that therapeutic strategies remain inadequate in treating the core symptoms of the disorder. In this respect, psychedelic medicine in combination with specialized psychotherapy represents a novel therapeutic strategy to treat disorders such as AN where treatment options have historically languished.

The purpose of this review is to highlight the potential suitability of psychedelic medicine for improving long-term treatment outcomes in AN, based on what is known about 5-HT signaling and cognitive function in patients and animal models. Although much of our argument is likely to be applicable to the therapeutic potential of other serotonergic or “classical” psychedelic compounds, including lysergic acid diethylamide (LSD) and ayahuasca, we focus here on psilocybin for three reasons: (1) the two FDA designations as a “breakthrough therapy” for treatment-resistant depression; (2) its prevailing use in numerous clinical trials for mental disorders; and therefore (3) the proximity to approval as the first widely used psychedelic in medicine and therapy. Further, we argue that fundamental research in animal models is necessary to reach a comprehensive understanding of the therapeutic effects of psychedelics in psychiatric disease. This is no different to the genesis and clinical acceptance of virtually every other pharmaceutical approach; however, in this instance the use of animal experimentation has the profound advantage of eliminating context and expectation that introduce confounds in the understanding of the neurobiological and pharmacological actions of psychedelic compounds.

## AN, 5-HT_2__A_ Signaling and Cognitive Flexibility

The genetic origins of AN have considerable overlap with other psychiatric disorders, most substantially with OCD (Yilmaz et al., 2018; Watson et al., 2019). These disorders also share phenotypes of cognitive dysfunction that reflect rigid rule-bound behavior and perseverative thinking (Bohon et al., 2019). AN and OCD have both been associated with a polymorphism in the 5-HT_2__A_ promotor (-1438G/A) (Enoch et al., 1998; Sorbi et al., 1998; Ricca et al., 2002; Walitza et al., 2002), the consequence of which is likely a loss of serotonergic function because patients with AN have reduced 5-HT_2__A_ binding compared to healthy controls in the frontal, parietal and occipital cortices (Audenaert et al., 2003). Reduced 5-HT_2__A_ binding in cortical regions persists after body weight recovery (Frank et al., 2002), indicating that diminished 5-HT_2__A_ receptor function is not simply a result of the acute effects of starvation. Several other disturbances in 5-HT signaling have been described as playing a role in the pathophysiology of AN, including alterations in both 5-HT_1__A_ receptor and 5-HT transporter binding in cortical and limbic structures (Bailer and Kaye, 2011) and reductions in cerebrospinal fluid concentration of 5-hydroxyindoleacetic acid (5-HIAA), the primary metabolite of 5-HT (Kaye et al., 1984). These points are consistent with the putative centrality of 5-HT in the genesis of the disorder, which begs the question; how do these neurochemical shifts in the brain manifest as behavioral outcomes? Cognitive inflexibility, linked to 5-HT signaling, is a prominent phenotype in AN and contributes to the persistent drive for weight loss despite the substantial negative consequences of starvation.

Cognitive flexibility is an executive function that includes attentional set-shifting, response inhibition, perseveration and reversal of stimulus-reward associations. Impaired cognitive flexibility has been demonstrated in healthy sisters of AN patients (Friederich and Herzog, 2011), and persists after body weight recovery (Tchanturia et al., 2012), suggesting it has etiological relevance for the development of the disorder (Roberts et al., 2010). While multiple cortical processes are likely to mediate perseverative tendencies and cognitive inflexibility in AN (Lao-Kaim et al., 2015), impaired set-shifting in AN patients is specifically associated with lower activity of the prefrontal cortex (Sato et al., 2013). While it is unclear whether cognitive inflexibility in AN is directly mediated by deficits in 5-HT_2__A_ receptor function, the finding that both AN and OCD patients share phenotypic features of behavioral rigidity as well as common genetic etiology involving 5-HT_2__A_ transporter function suggests that the two are correlated. In addition, the well-established potential for psychedelics to increase cognitive flexibility (Bouso et al., 2012, 2013; Carhart-Harris et al., 2016b) is consistent with the intriguing possibility that they may play a therapeutic role in addressing perseverative thinking and behavior in AN.

## The Neurobiological Actions of Psilocybin and Relevance to AN

Although it is well known that psilocybin, or more specifically its active metabolite, psilocin, acts as an agonist at multiple serotonergic sites, including the 5-HT_2__A_, 5-HT_1__A_, and 5-HT_2__*C*_ receptors (Halberstadt and Geyer, 2011; Tyls et al., 2016; Daniel and Haberman, 2017), focus has centered on the 5-HT_2__A_ receptor binding profile of psilocin due to the finding that specific 5-HT_2__A_ antagonists (e.g., ketanserin) block the majority of subjective effects of the drug in human subjects (Carter et al., 2007) and psilocybin-induced discrimination learning in mice (Winter et al., 2007). Psilocybin intake results in dose-dependent occupancy of the 5-HT_2__A_ receptor in the human brain, and both plasma psilocin levels and 5-HT_2__A_ binding are closely associated with subjective ratings of the intensity of psychedelic experiences (Madsen et al., 2019).

The other major neurobiological action of psilocybin administration is to decrease activity and functional connectivity within the DMN, a resting state network that is activated during higher-order cognitive tasks, such as considerations of past and future (Schacter et al., 2007), self-referential cognition (Northoff and Bermpohl, 2004; Sheline et al., 2009) as well as personal, social and moral judgments or decision-making (Spreng et al., 2009). Interestingly, resting state functional connectivity between the executive control network (ECN), a task-positive network primarily involved in cognitive control and emotional processing, and the anterior cingulate cortex is decreased in drug-naïve adolescents developing AN compared to healthy controls (Gaudio et al., 2015). During demanding cognitive tasks, the ECN typically shows increased activation, whereas the DMN shows decreased activation (Menon and Uddin, 2010). Moreover, the insula, a brain region that plays a critical role in switching between the ECN and DMN in divergent thinking (Sridharan et al., 2008; Heinonen et al., 2016) shows disrupted connectivity in patients with AN (Frank et al., 2016) (see Figure 1). Accordingly, it may be that psilocybin could act to shift cognitive processing away from an excessive internal focus and toward a more appropriate contextual balance. This style of information processing can more easily adapt to changing environmental needs, perhaps addressing aspects of cognitive inflexibility in patients with AN.

## Utilizing Animal Models to Interrogate the Neurobiology of AN

One compelling approach to understanding the etiology of AN is to take observations made in humans and rigorously dissect their underpinnings in experimental animal models where brain circuits can be perturbed and anorexic behavior can be interrogated. The most well-accepted animal model of AN, known as activity-based anorexia (ABA), exploits the innate motivation of laboratory rats to run in wheels. When rats with access to running wheels are placed on a restricted feeding schedule, there is a paradoxical increase in running activity despite substantially decreased caloric intake, causing a profound reduction in body weight (Gutierrez, 2013). It is important to recognize that within this model, food is only limited in terms of time, not quantity. With these same food access constraints and no running wheels, animals quickly learn to increase their food intake to maintain body weight. Similarly, given access to running wheels and free access to food, they maintain their body weight well. It is only the combination of timed availability of food and access to running wheels that initiates the precipitous reduction in body weight that typifies the ABA model. ABA is the only known model where non-human mammals choose self-starvation over homeostatic balance.

There are several behavioral and physiological similarities between ABA in rodents and human AN patients, including a predominance of phenotype in young females (Pirke et al., 1993), disrupted reward signaling (Foldi et al., 2017) and alterations in hormonal and neuropeptide function (Legrand et al., 2016; Scharner et al., 2018). Furthermore, several lines of evidence converge on the roles of reduced 5-HT signaling and cognitive inflexibility in the development of the ABA phenotype, and highlight the translational relevance of this model for examining cognitive deficits in human patients. 5-HT and its metabolites are shown to be maintained at lower levels in animals undergoing ABA compared to controls (Verhagen et al., 2009) and deficits in reversal learning have been shown in ABA rats (Allen et al., 2017). Other rodent studies implicate the direct impact of 5-HT function on cognitive flexibility, whereby rats lacking the serotonin transporter gene, which results in increased 5-HT availability at the synapse, show improvements in set-shifting and reversal learning paradigms, both measures of behavioral flexibility that are analogs of human cognitive flexibility tasks (Nonkes et al., 2012).

## Effects of Psilocybin in Animal Models

The behavioral effects of psilocybin in animal models appear to be mediated primarily through agonism of the 5-HT_2__A_ receptor, because the primary behavioral readout in response to psychedelics in rodents, the head-twitch response (HTR), is absent in mice lacking the 5-HT_2__A_R gene (Halberstadt et al., 2011). The other most frequent unconditioned behavioral responses to psychedelics in rodents are disruptions to sensorimotor gating (PPI) and exploratory behavior (Halberstadt and Geyer, 2018). Psilocybin also impacts associative learning in animal models by preventing rats from acquiring conditioned avoidance responses (Rambousek et al., 2014) and producing more rapid extinction of conditioned fear responses in mice (Catlow et al., 2013). In addition, psilocybin has been shown to induce dopamine (DA) release in the nucleus accumbens (part of the ventral striatum) and increase extracellular 5-HT levels in the medial prefrontal cortex in rats (Sakashita et al., 2015). This finding is tantalizingly consistent with the proposal that psilocybin could alleviate anorexic symptoms in the ABA model, not only because 5-HT signaling is reduced during ABA, but also because selective activation of DA projections to the nucleus accumbens prevents and rescues the ABA phenotype (Foldi et al., 2017).

Studies investigating the therapeutic potential of psilocybin in animal models of psychiatric disease have demonstrated significant alleviation of symptoms in mouse models of OCD (Sard et al., 2005) and post-traumatic stress disorder (PTSD) (Catlow et al., 2013), but did not significantly reduce anxiety phenotypes in wildtype rats (Horsley et al., 2018) or depression-related behavior in a genetic rat model, the Flinders Sensitive Line (FSL) (Jefsen et al., 2019). There are several reasons why the significant abatement of depression and anxiety symptoms in human clinical trials was not replicated in these animal studies. It may be that the behavioral test of depression in rodents, the Forced Swim Test (FST), does not fully capture the pathological state of major depressive disorder that is amenable to psilocybin treatment. The FST was originally developed as an antidepressant drug screen and aided the discovery of multiple drugs used successfully in the treatment of clinical depression. However, the behavior of rats and mice in this test is influenced by a number of factors including stress and activity levels, and immobility in this task has been suggested to reflect both adaptive behavior as well as despair (Molendijk and de Kloet, 2015; Anyan and Amir, 2018). Similarly, although FSL rats are shown to respond to traditional antidepressant medications, they also have markedly lower expression of 5-HT_2__A_ receptor mRNA in the frontal cortex (du Jardin et al., 2016), indicating that perhaps the 5-HT_2__A_-mediated effects of psilocybin may have a blunted response in these animals, driven by an inability to bind (Jefsen et al., 2019). This study did not examine the impact of psilocybin on brain function or receptor-specific activity, so whether depressive phenotypes in the FSL model correlate with altered 5-HT_2__A_ signaling remains to be resolved.

It is also likely that at least part of the impact of psilocybin on treatment-resistant depression in clinical trials is driven by the higher order subjective characteristics of psychedelic-assisted therapy and the known role of context in the efficacy of psychedelics (Carhart-Harris et al., 2018b). Indeed, certain subjective effects are shown to be critical for long-term clinical outcomes across multiple trials (Griffiths et al., 2016; Ross et al., 2016). Regardless, it remains the case that ethologically relevant animal models with specific neurobiological and behavioral features that are known to be amenable to the neurobiological impacts of psilocybin will provide unique insight into the therapeutic potential of psychedelics. One example is the ABA model, that displays both cognitive rigidity and disrupted 5-HT function analogous to the human condition. Such studies should test cognitive function in comparable ways to human cognitive test batteries, differentiate effects of single dose administration versus repeated dosing and investigate the impact of non-psychedelic 5-HT_2__A_ agonists to challenge the specificity of the effects of psilocybin on behavior and brain function.

## Summary and Conclusion

Recent clinical trials have put the spotlight on the therapeutic potential of psychedelics, specifically psilocybin, for a range of psychiatric disorders. The neurobiological and behavioral phenotype of AN, specifically with respect to modified serotonergic signaling and cognitive inflexibility, is well-positioned to be impacted by the putative effects of psilocybin treatment. New treatment strategies for AN are urgently needed, considering that up to 50% of patients with AN never recover (Pike, 1998), and the risk of death in patients with AN is more than five times higher than in the general population (Arcelus et al., 2011). Converging evidence suggests that psilocybin may be a promising novel treatment for AN, and well-designed trials including those in established animal models are warranted.

Surprisingly, beyond animal testing for safety and tolerability, the psychedelic research model has thus far largely bypassed the comprehensive screening of drug effects in animal models that usually punctuates the drug discovery pipeline to clinical trials in human patients. This may be based on the premise that psychedelic drugs will not impact animals in the same way as humans because of the uniqueness of the notable phenomenology in humans. Regardless, certain underlying neurobiological effects of psychedelics on cognitive function can be interrogated in animal models with much greater specificity than in human subjects. Animal models of disease also allow determination of the neurobiological effects of psychedelic drugs without the confounds of expectancy and bias that are impossible to control for in patient populations. They have been used successfully over many decades to enable rational pharmacology and the development of more targeted and efficacious medications.

The knowledge gained from animal studies of psychedelic medicines will add substantially to mechanistic accounts of clinical treatments for humans, and may afford improvements and innovations in clinical treatment. The important contribution that animal models can make in progressing the understanding (and possibly development) of psychedelic medicine as a viable therapeutic approach to some mental disorders is in the detailed scrutiny of brain function and behavior they provide. This aligns with a well-worn path whereby detailed mechanistic insights enable rational drug design. However, there is another less tangible benefit that may arise from a better, experimentally based understanding of the actions of psilocybin; reducing stigma associated with the use of psychedelics in a clinical setting. Across a wide range of community attitudes and social bias, it is increasingly recognized that education and understanding are key elements in dismantling stigma. Specifically, a reductionist understanding of the brain circuitry and neurochemistry that mediate the central effects of psychedelic medicines – independent of the confounds of human expectations and context – will help to demystify their clinical effects and hopefully diminish the prejudice associated with their use in the treatment of psychiatric disorders such as AN.

## Author Contributions

CF and BO contributed equally to the design, writing, and editing of the manuscript. PL and MW assisted in the revisions and editing of the manuscript.

## Conflict of Interest

The authors declare that the research was conducted in the absence of any commercial or financial relationships that could be construed as a potential conflict of interest.
